# An Essay on Diet in Health and Disease

**Published:** 1846-04

**Authors:** John Dawson

**Affiliations:** Jamestown, Ohio


**Art. II.**—*An Essay on Diet in Health and Disease.* **By John Dawson, M. D., of Jamestown, Ohio.**

Among the many inquiries of the physician, none, perhaps, is less free from perplexity, than the one which it is the design of this paper partially to discuss.

The proper diet of man, considered either in relation to health or disease, has been, and is, to some extent at the present time, surrounded with considerable confusion. Until recently the only test to which it had been with any success subjected, was the experience of different individuals upon their own persons or upon inferior animals. It is true, that reference, in order to determine the diet most suitable, has been frequently made to the habits of those uncultivated races living in the extreme North; and also to those living in the South, in the hope that something might be elicited that would minister to a more correct understanding of the subject. As yet, however, these imagined sources of information, although invoked with considerable industry, have failed to furnish facts of any other character than those of secondary importance. Nothing has been learned from the habits of the Esquimaux, Samoiedes, &c., who live exclusively on animal food, or from the natives of torrid regions, whose diet consists almost exclusively of the cocoa-nut, the plaintain, the banana, the sago, the yam, the cassava, the maize, and the millet; nor from the *ichthyophagi*, or fish eaters of antiquity; the *hylophagi*, that fed on the young shoots of certain trees; the *elephantophagi*, who lived upon the elephant; or the *struthiophagi*, whose diet consisted of the flesh of the ostrich, that goes to demonstrate the source from which a diet the most congenial to the moral, mental, and physical developments of man should be obtained. Surrounded as these different races and tribes have been, by a number of circumstances adequate to produce the intellectual and physical modifications by which they are distinguished, it becomes a question of no small importance, to know what that is to which their peculiarities should be attributed. The climate, the want of mental and moral culture, no less than their food, have evidently been instrumental in the production of some of the most distinguishing characteristics observed in the inhabitants residing in either of the climatic extremes. Who would say, that the small stature of the Samoiedes, their flat, round, broad face; thick lips; wide nose; little head; and black hair; together with their ignorance and superstition, are not as referable, to the frozen deserts they inhabit, which extends along the ocean forming the northern boundary of European and Asiatic Russia, as to the train-oil, and flesh of the rein-deer, upon which they almost exclusively subsist? Modification, physical or intellectual, therefore, under such circumstances, we can see no propriety in attributing exclusively to food. Most likely they are the result of a combination of causes all operating together. Indeed the animal food instead of being the cause of the diminished stature, &c., of the Esquimaux, is most probably made necessary by the nature of his climate. He needs the train-oil and other articles rich in carbon and hydrogen to furnish the materials of respiration, in order to maintain the heat of the body against the intense cold with which it is surrounded; hence the food of those in the extreme north may be regarded rather as a necessary consequence of their geographical or climatic position, than as a cause of the imperfections, moral or physical, that characterize them. The same might be affirmed concerning the food of the inhabitants of Borneo and Southern Hindostan. Leibig tells us that these people live on a diet containing only from 12 to 20 per cent. of carbon, and that this is made necessary and proper by the circumstances with which they are surrounded. Situated in an atmosphere highly rarified, and consequently more or less deficient in oxygen, and possessing a temperature but little higher than the air in which they live, they could not, it is supposed, enjoy health in any other way, than by living on the fruits in which their country abounds. The bacon and train-oil of the Laplander would be as little requisite to their comfort and health as would be his warm clothing.

The design of some things we sometimes ascertain by their construction. Cuvier was enabled to discriminate between carnivorous and herbivorous animals merely by an examination of their bones. The principle upon which his discrimination was founded, is that *each animal constitutes a whole, one systematic cycle; and that each part is exactly and invariably adapted to another, and is indicated by it.* By observing the integrity of this principle, he was enabled to classify fossil bones, arrange skeletons of individuals, species, and genera long since extinct. After being arranged, it was easy for C. to ascertain the class to which any given individual belonged; the habits, and kind of food upon which it was designed that it should subsist. Constituted with organs of motion for pursuing and overtaking prey, jaws articulated with great strength for carrying it off, teeth for tearing and dividing, claws for seizing, the animal was regarded as being carnivorous, designed by nature to live on flesh and blood. "In a word," says Cuvier, "the formation of the tooth indicates the structure of the jaw, and its kind of articulation; the structure of the shoulder bone, shows the form of the feet, just as the equation of a curve involves all its properties; and as by assuming each property separately as the base of a particular equation, we should reproduce both the ordinary equation and all its properties; so the nails and shoulder blade indicate the articulation of the jaw; the thigh bone and the other bones taken separately give the form of the tooth, or are given by it in their turn." Principles of a similar character have been applied to man in order to ascertain from his physical construction the kind of food most suitable to his nature. They show to the satisfaction of anatomists generally that he neither belongs to the one nor to the other, to the strictly herbivorous, or to the carnivorous; but that his organization designates him as occupying a rank that is intermediate. Twenty of his teeth, although comparatively small and but little adapted to grinding, resemble those of the herbivorous, while twelve bear conformations approaching those of the carnivorous animal. In the structure of his alimentary canal the same view seems to be confirmed. He neither possesses the complicated apparatus of the herbivora, the four stomachs of the ruminantia, (*osmasum*, *abomasum*, *ventriculus*, *reticulum*), nor the simple diminished tube of the carnivora. The articulation of the lower jaw with the glenoid cavity, as well as the muscles of mastication, are so strongly marked in the two great classes of animals that it is very obvious man belongs properly to neither. He possesses not in common with the carnivora, the deep glenoid cavity with the condyle or head of the jaw bone so firmly fixed in it as to make luxation impossible without great violence; nor the shallow, loose articulation, permitting the bone to be moved extensively in all directions, such as obtains with the herbivora. Natural facts of this character, when confirmed by others derived from other sources, are certainly entitled to some consequence in fixing the relations of man to the physical universe with which he is surrounded. Entitled to the station of even a minor grade of evidence, they favor the idea, if any thing can be learned from physical organization, that he is an *omnivorous* animal, designed to derive the articles inservient to his nourishment from either of the kingdoms; and that a mixed diet consisting of articles derived from both animal and vegetable matter would come nearer meeting the indications held out by nature than any other. Additional light has recently been thrown around this subject by the labor of organic chemists. A caption perhaps as good as any other in order to offer a few thoughts on this interesting and growing branch of science is,

*The Nutrient Elements of different articles of Diet.*—To organic chemistry many have looked with much confidence in the hope that something might be learned concerning the elements of nutrition that would at once settle the diversified views that have attached to the subject. For some time anterior to the labors of the organic chemists, it was suspected that the nutrient properties of all articles of diet, whether obtained from the animal or vegetable kingdom, consisted of a particular principle of a constant character. Haller, we know, supposed this to be jelly; Cullen thought it was an oily or saccharine matter; Stahl, Fordyce, Becker, and others imagined it to be a mucilage; Dumas, mucus; and Hallé regarded it as a hydro-carbonous oxide. Again, it was supposed that the organism performed the office of elaborating the peculiar principle destined for nourishment; that a change was wrought upon articles of diet by the gastric juice, under the influence of the vital force, before they could take any part in the nutritive function. The aspect of some of these views has undergone, of late, a change.

The gastric juice is now regarded as a substance in a state of metamorphosis, by the presence of which the food is merely dissolved. But the nutrient principles of the food, the organic elements subservient to nutrition are, as the ancients supposed, always the same—of a constant character, consisting of oxygen, hydrogen, carbon, and azote, arranged together in compounds denominated, in chemical nomenclature, *isomeric*, from the fact, that, although possessed of different physical properties, they have the same chemical elements united in the same proportions.

These nutritive principles, *albumen*, *fibrin*, and *casein*, as before remarked, are the same in chemical composition; and there is very good reason for supposing them to be one and the same substance. Denis succeeded in converting fibrin into albumen; that is, in giving to it the solubility and coagulability by heat which characterize the white of egg; and Mulder succeeded in showing that all three of the elements are nothing more than modifications of a certain body to which he gave the name of protein. This author found that "when animal albumen, fibrin, and casein are dissolved in a moderately strong solution of potash, and the solution is exposed for some time to a high temperature, these substances are decomposed. The addition of acetic acid to the solution causes in all three the separation of a translucent, gelatinous precipitate, which has exactly the same characters and composition from whichever of the three substances above mentioned it has been obtained." (Liebig's Animal Chem.)

What is very striking in regard to these proximate principles is, that they possess the same composition and the same characters whether derived from the animal or the vegetable kingdom. The following tables, which may be found in Leibig, confirm this position:

ANIMAL PROXIMATE PRINCIPLES, ACCORDING TO MULDER.

| | Albumen. | Fibrin. | Casein. |
|---|---|---|---|
| Carbon, | 54.84 | 54.56 | 54.96 |
| Hydrogen, | 7.09 | 6.00 | 7.15 |
| Nitrogen, | 15.83 | 15.72 | 15.80 |
| Oxygen, | 21.23 | 22.13 | 21.70 |
| Sulphur, | 0.68 | 0.33 | 0.36 |
| Phosphorus, | 0.33 | 0.36 | |
| | 100.00 | 100.00 | 100.00 |

VEGETABLE PROXIMATE PRINCIPLES, ACCORDING TO JONES AND SHERER.

| | Albumen. | Fibrin. | Casein. |
|---|---|---|---|
| Carbon, | 55 01 | 54.603 | 34.138 |
| Hydrogen, | 7.23 | 7.302 | 7.156 |
| Nitrogen, | 15.92 | 15.809 | 15.672 |
| Oxygen, Sulphur, Phosphorus, | 21.84 | 22.286 | 23.830 |
| | 100.00 | 100.000 | 100.000 |

Other organic elements there are, which according to chemical and physiological researches, do not take a conspicuous part in the process of nutrition; and hence are regarded rather as elements of respiration. Among these it is the present fashion to include fats, starch, gum, the different kinds of sugar, pectine, bassorine, &c. The first great class of principles properly, perhaps, styled the elements of nutrition, contain, as we have seen, azote; and on this account are called the *nitrogenized elements of food.* Those mostly concerned in sustaining respiration are called the *non-nitrogenized elements* or *elements of respiration.*

The speculations concerning the elements of nutrition may now be supplanted by the researches of a branch of science which proposes nothing else for consideration than demonstration,—demonstration by both analysis and synthesis.—These investigations show that the nourishing principles, albumen, fibrin, and casein, whether derived from the animal or vegetable kingdom are identical; exist already formed in vegetables and in the flesh and milk of animals; that, instead of being elaborated by some mysterious process from the articles taken into the stomach, they owe their existence to an agency altogether different. Animal albumen, fibrin, and casein, therefore, differing in no respect from the proximate principles of the same name derived from vegetable substances, it is obvious that it can make no difference from which class of substances food is obtained. That derived from animal possesses every thing in common with that from vegetable substances, and both agree in composition with these elements as found in the blood; that is, the albumen and fibrin of the blood are the same, as the albumen and fibrin found in animal or vegetable food.

*The proportion of Nutritious Matter in different Substances.*—Diversity obtains, doubtless, in the two great kingdoms of nature in regard to the manner in which the nutritious matters are found combined with adventitious substances that take no share in the nutritive process. Vegetable proximate principles, as a general rule, are found combined with a greater proportion of the elements of respiration, and also with a greater per cent. of redundant matter, answering, perhaps, no other purpose than that of keeping the bowels distended. This, although an important, and probably indispensable circumstance in the nutrition of animals strictly herbivorous, cannot be regarded as of any great moment in the nutrition of man. All experience teaches that graminivorous animals cannot live and thrive without *fodder*—something mixed with the food that operates mechanically in keeping the bowels and chyliferous ducts from becoming collapsed. Concerning man, whose digestive apparatus is differently constructed, this, in the present state of physiological science, cannot be so positively affirmed. He lives and thrives well on a diet containing a much less quantity of matter taking no share in the nutritive process.

Some of the results lately arrived at by *Boussingault* concerning the relative proportions of the nutrient principle found in substances usually consumed as food, are interesting. A principle well established in physiological science, and to which nothing is known to be opposed, is that increase of mass in the body, the development of its organs, and the supply of waste, are dependent upon the existence in the food of substances capable of being converted into blood; such, for example, as albumen, fibrin, and casein; the organic bodies from which fat is formed, and certain salts, particularly phosphate of lime, magnesia, and iron. From the indispensable necessity of such facts, as well as from a great number of experiments upon inferior animals, Boussingault lays it down as a general rule, concerning the proportions of nutritive matter found in different articles used as food, "*that the nutritious principles of plants and their products reside in their azotized principles; and consequently that their nutritious powers are in proportion to the quantity of azote they contain.*" Here is a table in which he contrasts the articles usually used as the food of man in reference to the azote they contain; and in explanation, says: "I have composed the following table or list of equivalents on this basis, (the proportion of azote contained) having assumed wheaten flour as the standard, and called it 100. As all herbs, roots, and leaves may be pulverized after dying, I have spoken of these articles dry under the name of meal."

| | | | |
|---|---|---|---|
| Wheaten flour, - - - | 100 | White-heart cabbage, - - | 810 |
| Wheat, - - - - - | 107 | Cabbage meal, - - - | 83 |
| Barley meal, - - - | 119 | Potatoes, - - - - | 613 |
| Barley, - - - - - | 130 | Potatoe meal, - - - | 126 |
| Rye, - - - - - - | 111 | Carrots, - - - - | 757 |
| Buckwheat, - - - - | 108 | Carrot meal, - - - - - | 95 |
| Maize, - - - - - | 138 | Turnips, - - - - | 1335 |
| Yellow peas, - - - | 67 | Mealy bananas, (Ticus Indica) | 700 |
| Horse beans, - - - | 44 | Manchat, (Casava plant) - | 700 |
| White French beans, - - | 56 | Name? (Discorea sativa) - | 300 |
| Rice, - - - - - - | 171 | Assio, (Anacacha) - - - | 1050 |
| Lentils, - - - - - | 57 | | |

From the data afforded by this table or list of equivalents, it appears that leguminous vegetables are possessed of a higher nutritive value than wheaten flour; tubers, including potatoes, carrots, turnips, and the seeds of the cereal grasses, are but moderately nutritious; rice, generally regarded as occupying a very conspicuous place in the list of highly nutritious substances, is partially deficient.

We are all aware, that countries are frequently quoted where vegetable articles, such as rice and the potato, constitute the sole articles of food with the inhabitants. From long experience in such countries, having lived among the inhabitants, Boussingault affirms such not to be the case. "In Alsace, for example," he says, "the peasantry always associate their potato diet with a large quantity of sour curdled milk. The same is true of the inhabitants of Ireland, who use large quantities of butter-milk or salt herring. The inhabitants of the Upper Andes, instead of living exclusively on potatoes, as travellers have asserted, use as their most common food '*lorco*,' a compound of potatoes and cheese. Rice, upon which it has been said the population of India almost exclusively subsist, appears always to undergo an admixture with other substances." Mr. Lequerri, after a long residence in India, says, "the food is almost entirely vegetable, and rice is the staple; the inferior castes only, ever eat meat. But all eat '*kari*,' an article prepared with meat, fish, or vegetables, which is mixed with the rice boiled in very little water. It is requisite to have seen the Indians at their meals to have any idea of the enormous quantity of rice they will put into their stomachs at a single meal. No European could cram so much at a time; and they very commonly allow that rice will not nourish them." (Boussingault on Rural Economy, and its relations to Chemistry, Physics, and Meteorology.)

Estimating the proportions of nutritious matter of different kinds of food by their chemical composition, and by experiments upon man and inferior animals, we shall fail in coming to any other conclusions than that *flesh*, of all the articles used as aliments, contains the least redundant matter, and is the richest in the materials necessary to sustain life and health. Playfair and Boeckman give for flesh, (fibrin, albumen, cellular-tissue and nerves) and for blood, one and the same formula, namely: carbon 48, nitrogen 6, hydrogen 39, oxygen 15. (Liebig's Animal Chem.) Liebig himself, says that 15 lbs. of flesh contain not more carbon than 4 lbs. of starch. When this matter is tested by experience it is found to be strengthened. Indirectly, the experiments of Magendie show, that animals, although incapable of living exclusively for any length of time on any one article, may, nevertheless, be supported for the greatest length of time on those organic principles having a composition identical with flesh. Liebig also tells us that the process of metamorphosis, and of nutrition, and reproduction goes on more slowly in graminivorous than carnivorous animals; and that the cause of this depends upon the fact that 4-5th lbs. of the food of the former are destitute of those nitrogenized compounds (fibrin and albumen) which go to make blood. A pig, according to him, fed with highly nitrogenized food, becomes full of flesh; fed with potatoes (starch) it acquires little *flesh*, but a thick layer of *fat*.

From these facts it is very probable that flesh, approaching as it does to the formula of blood, contains the greatest relative proportion of nutritive matter; and that, other things being equal, man can subsist on less quantities by weight of this article than any other known among the list of aliments. By no means do we intend to say that he can live in the enjoyment of health on flesh alone; or that this would be his proper food. But from the facts to which we have alluded, we regard the position as reasonable that it contains the greatest relative amount of nourishment.

While upon this part of our subject, it should not be forgotten, that some bodies highly nitrogenized, with others containing no nitrogen, although predominant in many articles used as aliments, fail when exclusively relied upon to sustain life, or even to maintain the system in a state of health. With the experiments of Magendie, Bostock, and Liebig, physiologists are familiar. By the first of these authors, it has long since been demonstrated, that sugar, olive oil, gum arabic, butter, pure wheaten bread, or rice, fail, when administered separately for any length of time to a dog, to sustain life. Phenomena, such as ulceration of the cornea, disappearance of fat, reduction of the size of the muscles, contraction of the stomach and intestines, an alkaline instead of an acid condition of the urine, too great a proportion of bilin or picromel in the bile, were observed to be the result, followed ultimately by death. Liebig also affirms that animals fed exclusively on gelatin, the most highly nitrogenized element of the food of the carnivora, died with the symptoms of starvation.

For this want of the nutritive power or principle, in the substances to which we have alluded, reasons are not easily assigned. Some of the animals, upon which experiments were made, were examined in order to see if the articles had undergone digestion. Magendie satisfied himself in relation to this point. He found that each of the substances had been converted into chyme, and that they afforded an abundant supply of chyle. Failing to find any thing in digestion adequate to an explanation of the subject, M. came to the conclusion, that *variety of diet* is an indispensable circumstance in this branch of hygiene—that the phenomena consequent upon his experiments were due equally as much to the simplicity of the food, as to any thing in relation to its properties. Stark, who sacrificed his life in making experiments of a similar character, came to the same conclusions. Upon this point, however, the labors of the organic chemist are useful. Mulder and Liebig have made the position very plausible, that the non-nitrogenized substances, such as sugar, starch, gum, the different kinds of *fat*, &c., are, properly speaking, the elements of *respiration*, rather than of *nutrition;* and that gelatin, although highly nitrogenized, is destitute of the proper arrangement in the proportion of its elements to form blood. It contains no sulphur or phosphorus, and less carbon, or more nitrogen than the organic elements from which blood is formed.

The fact should not be overlooked, in this place, that a source of fallacy, growing out of the habits of the animals experimented upon might have affected in a very material manner the results of Magendie's experiments. It could not be expected, for example, that a dog would thrive well on sugar, or that an ass could live on rice, or that man, accustomed to variety, could do well, for any length of time, on any single article. Personal observation is sufficient to convince any one, accustomed to the varieties of diet common to our climate, that it is impossible, without a sacrifice of health, to live for any length of time upon any one article that might be selected from the list of alimentary substances. Those acquainted with rural economy have always been familiar with the salutary results of variety in the feeding of domestic animals, and also with the deleterious consequences which result from a restricted system of regimen. At a manufacturing established in this State, for slaughtering sheep, the blood and inferior parts of the flesh were fed to hogs. It was found, as I have learned, that these animals, on such food, did not do well; were not easily fattened. Accordingly, meal was afterwards mixed with the food, and the result of this measure proved highly favorable. Now, without doubt, the effects of this experiment would have been different, had the animals been only accustomed to the digestion of animal food. Nevertheless, it would be very interesting to know which of the tissues, in the experiment, underwent the most alteration. The muscular, from its similarity to the food consumed, it might be supposed, suffered the least. Next in point of exemption from any great change were, most likely, those tissues composed principally of gelatin. Diminished condition of the nervous, pulpy, or medullary tissues composed principally of albumen united to a fatty matter, together with the deposition of fat, were probably the most conspicuous phenomena attending the experiment.

From this brief reference to facts, the accuracy of which cannot be called in question, without setting aside the only mode we have of testing the composition of organic bodies, it is very evident, that the greatest proportion of nutritious matter is found in animal substances, and other things being equal, animals of the inferior classes, not less than man, require, to enjoy health, variety of diet, and frequent changes from one article to another.

*Exclusive Vegetable-eating.*—Deference might make it proper in this place to advert, briefly, to the opinions of some persons concerning the propriety of exclusive vegetable eating. Frequently it is asserted that the nourishment of vegetables is better calculated to promote long life, a vigorous and healthy condition of all the functions, intellectual, moral, and physical, than that derived from animal matter. By such persons it is also asserted, that animal aliment was originally the main cause of human degeneracy; that it is discountenanced by the Bible; that it is calculated to make the individuals who use it vicious, intemperate, and blood-thirsty; that it gives rise to a great number of diseases, to which mankind would not otherwise be subject. Eulogies really the most passionate have been bestowed upon vegetable-eating, while reasonings the most elaborate have been directed against a mixed diet; and that too, not less by persons holding conspicuous stations in the commonwealth of letters, than by medical men, many of whom in their day were deeply versed in the literature of their profession.

Dr. Wm. Alcott's work is now before us with the following imposing title: "*Vegetable Diet as sanctioned by Medical men, in all ages.*" I find in this work such names as Rush, Cullen, Gregory, Lawrence, Cuvier, Home, Cheyne, Abernethy, and Muzzy, quoted in favor of man deriving his food exclusively from vegetables. In justice, however, to many of these distinguished authors it should be remarked, that they delivered themselves on this exclusive system with very considerable ambiguity. This is not true, I confess, of our friend Professor Muzzy, formerly of Hanover, New Hampshire, now of Cincinnati, Ohio. From the terms of his expressions he stands committed for a number of reasons, founded not less on moral than on physical considerations. Take, however, as a sample of ambiguity, the quotation from the eccentric Abernethy to show that *he* is in favor of the system:

"If you put improper food in the stomach it becomes disordered, and the whole system is affected. Vegetable matter ferments and becomes gaseous; while animal substances are changed into a putrid, abominable, and acid stimulus. Now, some people acquire preposterous noses; others, blotches on the face, and different parts of the body; others inflammation of the eyes, all arising from irritations of the stomach. I am often asked why I don't practice what I preach. I reply by reminding the inquirer of the parson and the sign-post—both point the way, but neither follows its course."

In relation to the various opinions which have obtained currency upon vegetable-eating, it may be submitted, that at the time that most of them were rife, the only certain method of testing the nutritive qualities of alimentary substances was in its infancy. Analysis of the animal and vegetable articles, commonly used as food, had not taken place. Until within the present century nobody had any evidence to believe that the nourishing principles were of a constant character;* always the same, whether derived from animal or vegetable

---

* We are aware, that, by some recent experiments, said to have been conducted with great care, Dumas and Cahourn are supposed to have succeeded in showing that the proportion of carbon is 7 per cent. less in fibrin than in albumen, while that of azote is from 8 to 9 per cent. more. These experiments will remove albumen and fibrin from the class of *isomeric* bodies. They do not, however, affect the question of the identity of animal albumen, fibrin, and casein with vegetable albumen, fibrin, and casein.

matter; and identical in chemical composition with the blood. Attested, nevertheless, as such facts have been by chemical analysis, they must have their influence in making up an opinion concerning the proper food of man. Experiments upon inferior animals strengthen their correctness. So, also, does a proper understanding of the process of chymification. Food when submitted to the action of the gastric juice is dissolved, but we are told that its elements, albumen, fibrin, and casein, are not changed in chemical composition. In his work on Animal Chemistry, Liebig tells us: "The clear gastric juice contains a substance in a state of transformation, by the contact of which with those constituents of the food, which by themselves are insoluble in water, the latter acquire in virtue of a new grouping of their atoms the property of dissolving in that fluid. During digestion the gastric juice when separated is found to contain a free mineral acid, the presence of which checks all further change. That the food is rendered soluble quite independent of the *vitality* of the digestive organs, has been proved by a number of the most beautiful experiments. Food, enclosed in perforated metallic tubes, so that it could not come in contact with the stomach was found to disappear as rapidly, and to be as perfectly digested, as if the covering had been absent; and fresh gastric juice, out of the body, when boiled white of egg, or muscular fibre was kept in contact with it for a time, at the temperature of the body, caused these substances to lose the solid form and to dissolve in the liquid." By this it appears that in chymification the elements of the food undergo no change except that of being dissolved, and made to assume a form ready for assimilation.

If then the constituents of the food derived from animal or vegetable matter be the same, and are not altered by chymification, but pass immediately into the blood unchanged, it is reasonable to suppose that considerations founded on something else than nutrition must operate on an individual who prefers vegetable to animal food.

Concerning the supposition that animal food makes those who use it vicious and sanguine in disposition, I have only to say, that such speculations seem more likely to have had their origin in the imagination, than in reason, or a careful and an extensive observance of the influence of food on the habits and dispositions of animals. Like causes under like circumstances, must give rise to something like the same effects. How, then, is it possible for animal, which as we have seen differs in no respect from vegetable food, to give rise to consequences which the latter is entirely incapable of producing.

Into a consideration of the *morality* of eating animal food we shall not enter. This question belongs to the department of Theology, and by the Divine Code only can it be settled. We shall now proceed to a slight consideration of a few points in relation to

*The Circumstances requiring a modification of Diet.*—

Only is it intended here to point out some of the circumstances that, *in health*, require a variation of diet. Hacknied as this part of our subject has been, in times past, it is still, perhaps, fertile enough to furnish employment for additional thought and experiment.

Among the circumstances to which we allude is the *season of the year* in which the diet is used. It is undoubtedly true that alimentary substances which might be proper at one season of the year may at another prove more or less injurious. Of course persons would do better on a diet calculated to promote calorification in winter, than in summer. In his theory of calorification, Liebig takes the very plausible position that the hydrogen and carbon of the food exercise an agency in the production of animal heat. It matters not, he informs us, what intermediate changes the food may undergo in the organism, the last change is the conversion of its carbon into carbonic acid, and its hydrogen into water. The oxygen for these chemical processes is taken in chiefly through the lungs; and the author to whom we have alluded, contends, that, inasmuch as oxygen cannot combine with either hydrogen or carbon *outside* of the body without the liberation of heat, therefore it is rational to suppose that the same processes cannot go on *inside* of the body without being attended with a corresponding result; and also, that the quantity of heat is just the same as if the carbon and hydrogen had been burned in the open air. If such positions be correct; if they are to be regarded as the literature of calorification at the present time, then all must agree that food rich in carbon and hydrogen suits better for winter than for summer. The habits of nations on different parts of the globe are referred to by Liebig in order to strengthen the positions advanced.

In the South we are told the natives prefer to feed on the fruits which in the fresh state contain not more than 12 per cent. of carbon; while those in the Arctic regions live principally on bacon and train-oil containing from 60 to 80 per cent. of carbon. In temperate climates, it is thought, we need about one-eighth more carbon in winter than in summer.

Recently we have had some additional testimony on this subject. In a paper by John Robertson, of Manchester, England, "*On the period of Puberty in Esquimaux Women*," a letter is published from the Rev. J. Lundberg, Superintendent of the Moravian Missions, in which we have the following facts: "We observe with regret that the now more frequent use of European provisions, as bread, flour, peas, &c., (instead of fish and flesh) acts injuriously on the bodily constitution of the Esquimaux. They become weaker and are attacked with some diseases which were formerly unknown in Labrador."

Bearing indirectly on this matter is the fact, that animals eliminate more carbonic acid by the function of respiration at a low, than at a high temperature; more, therefore, in winter, than in summer. F. M. Letellier, lately in a memoir to the French *Academie des Sciences*, related some experiments proving that the amount of carbonic acid expired at 32° Fahr. was about twice as much as at the temperature of 86°. For the production of this relative excess of carbonic acid, the food certainly must contain an excess of carbon; because it is axiomatic that the excretions during health can contain nothing not previously taken into the system, either as food or as the materials of respiration. If, then, there be a connexion between the constituents of the food and the production of animal heat, it is obvious that modifications of the former are indispensable to a physiological regulation of the latter. Now, whether we adopt the theory of *Lavoisier*, *Seguin*, and *Liebig*, that the heat of the system is derived from the combination of the oxygen inspired, with the carbon and hydrogen of the metamorphosed tissues which proceed ultimately from the food; or that of *Black*, that it is the result of the latent heat of the inspired air becoming sensible in the lungs; or that of *Brodie*, that it is derived from the great nervous centres; or that of *Chosat*, who thinks that the trisplanchnic nerve is the great organ for its development; or that of *Home*, that it depends on the ganglionic part of the nervous system; or that of *Phillip*, who regards it as a secretion; or that of those who look upon it as the joint production of the three great functions of life, innervation, circulation, and secretion,—certain it is, that the theory of combustion as defended and explained by Liebig, is the most philosophical of any that has yet been proposed. Resisted, indeed, the positions cannot be that a union of oxygen with hydrogen and carbon, giving rise to water and carbonic acid, takes place some how or other in the system; and that these combinations cannot take place without the liberation of constant quantities of heat, as much as if burned in the open air, or oxygen gas. The circumstances then being present, it is rational to conclude, that, although under the controling influence of the vital force, this force is only exerted in placing the elements in a proper condition for that play of the affinities by which the phenomena of heat are produced.

It has for some time been known, that the relative proportion of oxygen in the atmosphere is greater in winter than in summer; and consequently that the amount inspired is greater at the former than at the latter period. This excess of oxygen corresponds with the excess of carbonic acid evolved during cold weather, as shown by the experiments of Letellier. Now, if there is taken into the system in a low temperature of the atmosphere a relative excess of oxygen; and if there is a relative excess of carbonic acid during the same period expired, how can we resist the conclusion that the food must contain carbon in quantities sufficient for the conversion of the excess of oxygen into carbonic acid? Does not also the production of this excess of carbonic acid enable us to understand how it is that the inhabitants of the polar regions are enabled to resist a temperature from 70° to 90° lower than that of their bodies?

Taken then as sustained, that the diet should correspond with the temperature of the atmosphere in the different seasons of the year, it might now be inquired what kind of diet is best calculated to promote health. Imperfectly, we are aware, in the present state of our knowledge, can this question be answered. There can be but little doubt, that the practices, which have obtained among the inhabitants of the climatic extremes and temperate latitudes, are in the general correct; such as are demanded by the peculiar conditions of the atmosphere in which they reside. Those inhabiting the arctic regions will enjoy the best health by using food rich in hydrogen and carbon, such as the oleaginous substances. In the South, of course, the diet just mentioned would be contra-indicated. Fruits and such articles as are deficient in the elements of calorification, with them would be best calculated to promote health. We of a temperate climate, it might reasonably be presumed, should make use of a diet approaching in winter that used by the inhabitants of the North; while in summer it should correspond more nearly to that used by the inhabitants of the South. This is exactly the practice, which so far as we are acquainted in our climate has pretty universally obtained. We consume mostly during winter the oleaginous parts of animal food, such as bacon, fat meats of all kinds, and butter; while in summer the diet consists more of the vegeto-gelatinous, the saccharine and the farinaceous; the former class rich in carbon, the latter partially deficient.

Of the influence of *age* in requiring modifications of diet, every one, the least conversant with dietetics is familiar. From the first hour of infancy until adult age, and from this until the last periods of decline, the system is undergoing continual mutations; and the requirements of each period must obviously correspond with the different conditions of the system.

In childhood where respiration is very frequent (35), the circulation quick (120), and the temperature high (102°,) nature has made a provision in the form of milk, which is a natural product perfectly fitted to sustain life. Analytically considered, this substance is found to contain all the constituents requisite for the development and growth of the young system. The casein of which it is in part composed, is a nitrogenized constituent, distinguished from fibrin and albumen by its great solubility, and by not coagulating when heated. It is identical in composition with the blood, containing, however, a much larger proportion of the earth of bones, and that in a form so soluble, as to be capable of reaching every part of the body. Milk also contains sugar and butter, substances having no nitrogen in their composition, but large proportions of carbon and hydrogen. Regarded as a compound, there appears to be, in the composition of milk, an obvious adaptation of means to ends. For the development and continual growth of the bones, muscles, &c., the casein is provided; and, to answer the wants of the exalted temperature and the frequency of the respirations, materials abounding in carbon and hydrogen are found in the butter and sugar.

Very *old persons* have a condition of the system that differs widely from that incident to childhood. With them waste and supply are not in a state of equilibrium. The former, in a normal condition of the system predominates. Ordinarily in *confirmed old age* or *caducity,* there is noticed among other changes incident to this period, an atrophied state of the thorax and lungs, which necessarily diminishes the amount of oxygen inspired; predispositions of the voluntary and involuntary muscles to paralysis, from want of nervous influence; ossification of the valves of the heart and arteries; torpidity of the chylopoitic apparatus; and an inefficient condition of the function of calorification. A diet suitable to these various states of the organism, must differ widely from that proper to a child, or to an individual in the period of adolescence. The casein of milk contains too great a proportion of the earth of bones. Such constituents are not needed, as the osseous system is already developed. Butter contains hydrogen and carbon in proportions too great to be supplied with their equivalents of oxygen. Diminished so much in capacity, the lungs, it would be reasonable to suppose, would fail in furnishing the requisite quantities of oxygen for the oxidation of these elements. To most observers it is also known that, not only butter, but fats of all kinds, are digested with difficulty. Such being the case they are, in consequence of the weakness of the digestive organs of an old person, contra indicated. To keep the system in a physiological condition the food, it might be presumed, should consist, in order to supply waste, of articles containing a reasonable proportion of nutritious matter, and also of a sufficiency of elements calculated to maintain the function of calorification. For these purposes the most digestible kinds of flesh, farinaceous, and the vegeto-gelatinous substances, all things considered, would, perhaps, answer as well as any other to fulfil the indications presented. Leguminous plants, it should be recollected, are of all vegetable substances the richest in nutritive principles, and from such a consideration would likely be better adapted to the condition of the system we have been considering than flesh of any kind. Old persons while in the enjoyment of apparent health are found to suffer more than any other class from the effects of a low temperature of the atmosphere. My own observations in relation to them are, that the most of them die during winter; and often too, without any other visible cause than coldness of the weather. Many indeed, go to bed at night seemingly well, but in the morning are found dead. Unqestionably the cause of all this will find its best explanation in the imperfect condition of the function of calorification; and as a consequence the food, no less than other things, may reasonably be invoked in trying to avert such a catastrophe.

Between the two extremes we have the period of *virility* or manhood, in which the system differs from that of an old person in the fact that there is no preponderance of the various functions in favor of *waste;* and from the child in that there is none in favor of increase of mass. Waste and supply are exactly balanced; and notwithstanding the immense amount of substances taken into the system during a year (there is of oxygen alone 746 lbs., according to Lavoisier, and of carbon 5,073 ounces,) by the assimilative and respiratory functions, the body at the end of that time is found, as regards its weight, not to be increased. With such an individual we should not expect a diet to agree unless it comported with the peculiar condition of the system. To increase the mass and build up the osseous system, as in the case of a child, a peculiarity of substances alimentary would not be needed. Nor would the diet require to be shaped in accordance with the waste and lack of energy found in the principal functions of life in an old person. Food calculated to keep waste and supply balanced, and to afford the necessary materials of respiration would seem to be what is indicated. Best adapted to the accomplishment of these ends, are, as we have elsewhere shown, those nitrogenized substances abounding in albumen and fibrin, and also the non-nitrogenized substances. We have seen that the former are contained in greatest proportions in flesh, the leguminous plants, lentils, and wheaten flour; the latter in the various fats, oleaginous substances generally, potatoes, maize, &c. As there are, however, a number of circumstances which require that the food should be varied, and without due respect to which all the blessings of diet may be frustrated, we shall now advert to some of these, but only to give them a very partial consideration.

Prominently among these are *occupation* and *habit.*

The man engaged, from 12 to 16 hours every day, in physical efforts, may use a diet with impunity, nay with advantage, that would cause disease in a person engaged in intellectual pursuits, connected with confinement and sedentary habits. Strikingly is this fact exemplified in every day's experience. The man at hard labor during the day is able to consume at night large portions of flesh, fat, vegetables, &c.; while another one, sitting at the writing table all day, makes a very good meal on two or three biscuits and a cup of tea. Both enjoy good health, but it is only in consequence of employing such alimentary substances as are suitable to their occupations. The physician actively engaged in the arduous duties of his profession knows that he can then digest articles in quantities and of qualities, that under other circumstances would operate upon him as a source of great annoyance. Satisfactory reasons for this are easily assigned. An individual at labor respires more frequently than when at rest; the circulation is carried on with greater rapidity; the gastric juice is secreted more copiously, and indeed, increased activity applies, not less to all the secretory organs, than it does to those the office of which is to excrete. By the frequency of respiration the amount of oxygen introduced into the system is greatly augmented, and as a consequence the constituents of the venous blood arising from the metamorphosis of the tissues are rapidly oxidated. The augmented condition of the circulation quickly displaces the effete parts of organs and tissues; and hence there is created in the system, in order to supply their place, an absolute demand for substances in quantities sufficient for the purposes of assimilation. An increased supply of gastric juice corresponding with the other conditions present in the system of a laboring man is an indispensable circumstance, and favors the digestion and assimilation of his food. At rest, with no food in the stomach, the gastric juice is secreted in small quantities. The reverse of this takes place when the stomach is full and there is exercise. Here then are present all the circumstances necessary to the digestion of the largest quantities of food.

With a person engaged in intellectual pursuits a different state of things obtains. The severe student or author knows that after indulging in a full meal of stimulating animal or vegetable food, his mind is rendered dull, stupid, and inactive; and is incompetent to the performance of its ordinary labor. A singular indescribable sensation of uneasiness, attended with indisposition to bodily or mental exertion pervades the entire system; and, as a matter of course, he is compelled to remain idle during the hours of digestion. The cause of this trouble has generally been sought for in the supposition that it is all occasioned by the presence of the food withdrawing the nervous and vascular influence from the brain and colonizing them for a time in the stomach. Good as this reason appears to be, it may be made better by connecting it with the fact, that the functions of secretion and excretion, of circulation and respiration in the intellectual man, if not impaired, are usually below the standard of health. Generally, intellectual men are observed to have pale faces, weak circulation, little muscular strength, and an enfeebled condition of the digestive organs. Taken all together, therefore, these things do much towards showing the propriety of such variations in the food as will comport with the peculiarities of the organism. Incidentally it might here be remarked, that the making of a proper selection of alimentary substances, by men engaged in intellectual pursuits, is of the utmost possible importance. Without a strict attention to his diet no man can make progress in intellectual labor. The right exercise of the mind is to a certain extent dependent upon the stomach. When the stomach contains substances in quantities, or of qualities that it cannot readily digest, a suspension of all the energies of the mind is the consequence. This, however, is not all; the stomach, even after the load from it is removed, is for some time afterwards in a disordered condition. The repetition of such errors soon leads to disease, by which the individual is often converted into a confirmed invalid. By no means do we wish to be understood that men who labor with their minds should not eat; nor would we even say that they should not regard the consumption of the luxuries of our climate as a source of enjoyment, and thus, if they think proper, make the saying good, that they "*live to eat.*" There is a diet proper for them, as well as for laboring men. But it has its peculiarities, and it is these, we urge, that should be made the subject of the strictest attention during life. As we have partially alluded, in another place, to the diet most proper, and as every individual has his own idiosyncracies that should invariably be consulted, we shall not insist further upon the subject than by adverting to a maxim of former days, which is, that "so far as his food is concerned, man is either his own physician or a fool at the age of forty."

That *habit* has something about it that should be consulted in the selection of a proper diet, seems pretty obvious. According to vulgar expression, it is "*second nature.*" We see this strikingly manifested in individuals who in the early period of life and while the organism is being developed, are accustomed to active exercise or hard labor; but at the period of manhood, or afterwards, adopt habits of a contrary character. Such find that without making a corresponding change in diet, or some regulations in regard to exercise, they are precipitated into disease. I have often noticed this to be the case with young students. Many of them are accustomed in early life to active exercise or labor. More or less of this they usually take during their private pupilage. When, however, they go to attend the teaching of professors, their former habits ar forgotten. Duty requires a large portion of their time in their rooms, or at the college; and the result is disease of the digestive organs, and impairment of the secretions generally of the system. It has been supposed that active exercise in early life exerts a favorable influence in developing and hardening the physical powers. This is very true, but it also creates a demand in the system for the continuance of such habits. In consequence of this, persons accustomed to labor in early life, bear the confinement incident to intellectual pursuits with much more inconvenience, than those who have spent the first periods of life in comparative idleness. Besides students proper, there is another class of persons among whom the annual mortality in our country from want of proper dietetic regulations is very high. I allude to those who having been raised to habits of active exercise or labor, adopt afterwards, as a means of obtaining a livelihood, some sedentary mechanical pursuit, or perhaps that of standing behind the counter in mercantile establishments. In a short time such find the appetite failing; grow pale from a sluggish condition of the circulation; are afflicted with constipation, sour stomach, gasterrhœa, &c. Instead of regarding these inconveniences as results of a change of habit, which could be easily remedied, recourse is too often had to stimulants, sweet-meats, and trash of various kinds, than which nothing could exercise consequences more injurious. The only way then by which all those who have been accustomed to active exercise can avoid much inconvenience and disease, is by strict regard to regimen, and such other things as will comport with their change of habit and occupation.

[TO BE CONTINUED.]

